# Thymoquinone Lowers Blood Glucose and Reduces Oxidative Stress in a Rat Model of Diabetes

**DOI:** 10.3390/molecules26082348

**Published:** 2021-04-17

**Authors:** Mohamed Faisal Lutfi, Abdel-Moneim Hafez Abdel-Moneim, Ashwag Saleh Alsharidah, Mugahid A. Mobark, Ahmed A. H. Abdellatif, Imran Y. Saleem, Osamah Al Rugaie, Khalid M. Mohany, Mansour Alsharidah

**Affiliations:** 1 Department of Physiology, College of Medicine, Qassim University, Buraydah 51452, Saudi Arabia; mf.lutfi@qu.edu.sa (M.F.L.); a.elmonem@qu.edu.sa (A.-M.H.A.-M.); ashriedt@qu.edu.sa (A.S.A.); 2 Department of Physiology, Faculty of Medicine, Nile College, Sheikh Zayed 7121, Sudan; 3 Department of Physiology, Faculty of Medicine, Mansoura University, Mansoura 35516, Egypt; 4 Department of Pharmacy Practice, College of Pharmacy, Qassim University, Mansoura 51452, Saudi Arabia; mu.mohammed@qu.edu.sa; 5 Department of Pathology, Faculty of Medicine, University of Kordofan, El-Obeid 13314, Sudan; 6 Department of Pharmaceutics, College of Pharmacy, Qassim University, Buraydah 51452, Saudi Arabia; A.Abdellatif@qu.edu.sa; 7 Department of Pharmaceutics and Industrial Pharmacy, Faculty of Pharmacy, Al-Azhar University, Assiut 71524, Egypt; 8 School of Pharmacy & Biomolecular Sciences, Liverpool John Moores University James Parsons Building, Liverpool L3 5UG, UK; i.saleem@ljmu.ac.uk; 9 Department of Basic Medical Sciences, College of Medicine and Medical Sciences, Qassim University, Unaizah, P.O. Box 991, Qassim 51911, Saudi Arabia; o.alrugaie@qu.edu.sa; 10 Department of Medical Biochemistry and Molecular Biology, Faculty of Medicine, Assiut University, Assiut 71515, Egypt; khalidmohany@aun.edu.eg

**Keywords:** diabetes mellitus, glycaemic control, lipid profiles, oxidative stress, renal function, thymoquinone

## Abstract

The aim of the present study was to assess the short-term effects of Thymoquinone (TQ) on oxidative stress, glycaemic control, and renal functions in diabetic rats. DM was induced in groups II and III with a single dose of streptozotocin (STZ), while group I received no medication (control). The rats in groups I and II were then given distilled water, while the rats in group III were given TQ at a dose of 50 mg/kg body weight/day for 4 weeks. Lipid peroxidase, nitric oxide (NO), total antioxidant capacity (TAC), glycated haemoglobin (HbA1c), lipid profiles, and renal function were assessed. Moreover, the renal tissues were used for histopathological examination. STZ increased the levels of HbA1c, lipid peroxidase, NO, and creatinine in STZ-induced diabetic rats in comparison to control rats. TAC was lower in STZ-induced diabetic rats than in the control group. Furthermore, rats treated with TQ exhibited significantly lower levels of HbA1c, lipid peroxidase, and NO than did untreated diabetic rats. TAC was higher in diabetic rats treated with TQ than in untreated diabetic rats. The histopathological results showed that treatment with TQ greatly attenuated the effect of STZ-induced diabetic nephropathy. TQ effectively adjusts glycaemic control and reduces oxidative stress in STZ-induced diabetic rats without significant damaging effects on the renal function.

## 1. Introduction

Diabetes mellitus (DM), a chronic metabolic disorder that is prevalent in humans, is associated with abnormally high levels of glucose in the blood. Elevated glucose levels increase oxidative stress [1] by stimulating various mitochondrial enzymes, which results in the overproduction of reactive oxygen species (ROS) [2] and has detrimental effects on many organs [3]. Mullarkey et al. [4] reported multifactorial aetiologies of the augmented levels of oxidative stress associated with DM. Recent studies have shown that β-cell dysfunction in diabetic patients is mostly the result of enhanced oxidative stress [1,5]. Other conditions associated with DM are hypertriglyceridemia, low levels of high-density lipoprotein (HDL), and high levels of low-density lipoprotein (LDL) [6], all of which contribute significantly to the development of atherosclerosis [7]. When exploring the efficacy of anti-diabetic drugs, having antioxidant effects [8,9], efficacy in correcting dyslipidaemia [10] and hyperglycaemia [11], as well as safety, are crucial.

Current diabetes treatments include insulin and a number of oral antidiabetic drugs with severe adverse effects; thus, it remains a challenge to manage diabetes without any side effects. The search for more efficient and safe hypoglycaemic agents has therefore remained an important research area [12]. Thymoquinone (TQ), the main volatile constituent of oil from *Nigella sativa* seeds, has been shown to have a broad therapeutic spectrum [13,14,15,16], and studies on the potential effects of TQ on oxidative stress, β-cell damage, and diabetic nephropathy are promising [17,18]. TQ exhibits protective effects against diabetes, inflammation, and oxidative stress. The antioxidant and anti-inflammatory activities of TQ may cause its clinical effect against various diseases. It penetrates physiological barriers and accesses subcellular compartments and exhibits the radical scavenging effects. It also reacts with glutathione (GSH), NADH, and NADPH to form glutathionyl-dihydro-TQ (reduced species) and combats free radicals [19]. TQ can be effective in persons with glucose intolerance as it helps to reduce appetite, glucose absorption, hepatic gluconeogenesis, blood glucose, cholesterol, triglycerides, and body weight. It simulates glucose-induced insulin secretions from pancreatic beta cells and improves glucose tolerance as effectively as metformin [20]. Megantara et al. [21] confirmed the antidiabetic activity of TQ against pioglitazone. Al-Ali et al. [22] established the safety of TQ administered orally to experimental animals through determining the lethal dose (LD50) of TQ both in mice and rats. Asgary et al. [23] reported that TQ is safe and well-tolerated with no severe side effects. If short-term therapy is the best treatment method, then this could result in a reduction of waitlists and thus a better entrance to evidence-based care [24].

The aim of the present study was to evaluate the short-term effects of TQ on oxidative stress, glycaemic control, lipid profile, and renal function.

## 2. Material and Methods

### 2.1. Materials

Streptozotocin (STZ), TQ, and total glycated haemoglobin kits were purchased from Sigma–Aldrich (St. Louis, MO, USA). Lipid peroxidation (LPO) assay kit, total serum nitrate/nitrite (NO) kit, total antioxidant capacity (TAC) kit, and serum creatinine and urea concentrations kits were purchased from Biodiagnostic (Cat. Tahrir, Cairo, Egypt). Triglycerides assay kit, total cholesterol assay kit, and HDL and LDL precipitating reagent kits were purchased from United Diagnostics industry (UDI, Makka, KSA). All chemicals used were of analytical grade and were used as received without any further purification. All solutions were prepared with Millipore water. 

### 2.2. Ethics

This study was approved by the Subcommittee of Health Research Ethics, Deanship of Scientific Research, Qassim University (Approval No: 18-01-09), in accordance with the National Research Council (US) Guide for the Care and Use of Laboratory Animals [25].

### 2.3. Animals

The present study used 18 male Sprague-Dawley rats, weighing 200–250 g. The rats were maintained at a temperature of 24–25 °C and a humidity of 50–80% under a 12:12 h light:dark cycle with free access to standard rat pellets and water throughout the experiments. The animals were treated according to the principles outlined in the NIH Guide for the Care and Use of Laboratory Animals [26]. Rats were obtained from the animal house of King Saud University/Kingdom of Saudi Arabia (KSA), acclimatised to the laboratory conditions at 25 °C for one week and fed a standard diet with water ad libitum at College of applied medical sciences (CAMS)/ Qassim University/KSA.

### 2.4. Induction of Diabetes

The study included three equal groups of rats (N = 6 rats for each group). DM was induced in groups II and III with a single dose (65 mg/kg body weight) of STZ that was freshly dissolved in 5 mM citrate buffer, pH 4.5 [27]. Control animals (group I) were injected with an equal volume (1 mL) of the buffer solution alone. Fasting blood glucose levels (BGLs) were assessed with a glucometer (Accu-Chek Active, Roche Diagnostics, Germany) two days after the injection of STZ. Rats were considered diabetic if the BGL was greater than 200 mg/dL [28]. Subsequently, all animals were maintained for 4 weeks on ad libitum food and water with checking of fasting blood glucose, average body weight, and food and water ingestion before the beginning of treatment with TQ [19].

### 2.5. Administration of the Experimental Drug

The control rats in group I were healthy rats. The rats in group II (untreated diabetic group) received no medications. Both groups I and II were given 1 mL of distilled water per 100 g of body weight/day by oral gavage for 4 weeks. The rats in group III were given TQ, which was initially dissolved by adding dimethyl sulfoxide (DMSO), followed by normal saline (for a final DMSO concentration of less than 0.5%). The resulting TQ solution was administered at a dose of 50 mg/kg of body weight once daily using gastric gavage for up to 4 weeks [19].

#### 2.5.1. Sample Collection

Rats were anaesthetised with ether and sacrificed at the end of the experimental period (4 weeks). Blood samples were collected from the medial canthus of the eye via heart puncture into sterilised tubes for serum separation, while whole blood was used to assess total glycated haemoglobin (HbA1c) [29].

### 2.6. Total Glycated Haemoglobin

Total glycated haemoglobin was assessed with a total glycated haemoglobin kit (Sigma–Aldrich, St. Louis, MO, USA) according to standard techniques adopted by Bunn et al. [30].

### 2.7. Oxidative Stress Assays

#### 2.7.1. Lipid Peroxidation (LPO)

The state of LPO was assessed colorimetrically with a lipid peroxide kit (Biodiagnostic, CAT. NO MD 2529, Egypt) according to Ohkawa et al. [31]. Using this colorimetric method, thiobarbituric acid (TBA) reacts with malondialdehyde (MDA) in an acidic medium at a temperature of 95 °C for 30 min to form reactive TBA products. The absorbance of the resulting (pink) product was measured at 534 nm with a lipid peroxide kit (Biodiagnostic, CAT. NO MD 2529). The values are expressed in nM.

#### 2.7.2. Total Antioxidant Capacity (TAC)

TAC was measured colorimetrically with a TAC kit (Biodiagnostic, CAT. NO TA2513, Egypt), according to Koracevic et al. [32]. Using this method, antioxidant capacity was analysed via a reaction between the antioxidants in the sample and a known amount of exogenous hydrogen peroxide (H_2_O_2_). Antioxidants in the sample eliminate a portion of the hydrogen peroxide, and the residual H_2_O_2_ is measured colorimetrically via an enzymatic reaction that converts 3,5-dichloro-2-hydroxybenzenesulfonate to a coloured product, which is subsequently measured at 505 nm. The values are expressed in mM/L.

#### 2.7.3. Determination of Total Serum Nitric Oxide (NO)

The levels of NO were represented by total nitrite concentrations using a colorimetric method called the Griess reaction. The colour—developed by reacting with the Griess reagent (1% sulphanilamide/0.1% naphthylethylenediamine dihydrochloride, *w*/*v* in 2.5% H_3_PO_4_)—was recorded at 550 nm against a reagent blank using 10–100 µM sodium nitrite as a standard [33]. The values are expressed in nmol/mL.

### 2.8. Assessment of Serum Creatinine and Serum Urea

#### 2.8.1. Serum Creatinine

Serum creatinine was measured colorimetrically with a creatinine kit (Crescent Diagnostics, CAT. NO CS604, KAS) using the kinetic Jaffe reaction (i.e., without deproteinization) [34]. In the Jaffe reaction, creatinine reacts with an alkaline precipitate to produce a reddish orange colour, the intensity of which is directly proportional to the creatinine concentration at 490 nm. The values are expressed in mg/dL.

#### 2.8.2. Serum Urea

Serum urea was measured using a urea kit (Crescent Diagnostics, CAT. NO CS612, KSA) using an enzymatic, colorimetric endpoint (i.e., the Berthelot method) [35]. In this reaction, urease catalyses the conversion of urea to ammonia. In a modified Berthelot reaction, ammonia ions react with a mixture of salicylate, hypochlorite, and nitroprusside to yield a blue–green dye (indophenol). The intensity of this dye, which is measured at 640 nm, is proportional to the concentration of urea in the sample. The values are expressed in mg/dL.

### 2.9. Lipid Profiles

#### 2.9.1. Triglycerides

A triglycerides assay kit (CAT. NO EL59L-1000, KSA) was used to measure serum triglycerides using the enzymatic-colorimetric glyceryl phosphate oxidase method [36]. The values are expressed in mg/dL.

#### 2.9.2. Total Cholesterol

A total cholesterol assay kit from UDI (CAT. NO EL24-1200, KSA) was used to measure total cholesterol via an enzymatic, liquid, colorimetric test called the cholesterol oxidase phenol 4-aminoantipyrine peroxidase (CHOD/PAP) method [37]. The values are expressed in mg/dL.

#### 2.9.3. HDL Cholesterol

A kit from UDI (CAT. NO EL41-360, KSA) was used to measure HDL via the enzymatic-colorimetric phosphotungstate method [38]. Using this method, LDLs and very low-density lipoproteins (VLDLs) are precipitated out when the serum reacts with the precipitating reagent, while the HDL fraction remains in the supernatant. The supernatant is then used as a sample in the cholesterol assay. The values are expressed in mg/dL.

#### 2.9.4. LDL Cholesterol

LDL was measured indirectly based on the levels of total cholesterol, HDL, and triglycerides using the Friedewald formula [39]:

LDL=Total cholesterol−HDL−(Triglycerides/5)

The values are expressed in mg/dL.

### 2.10. Histopathological Evaluation of the Kidneys

The kidneys from each animal were sliced and fixed in 10% buffered neutral formalin. The formalin-fixed tissues were dehydrated and infiltrated using an automated tissue processor (Leica TP1020) and embedded in paraffin wax. Serial 5 µm thick sections were prepared using a microtome (Leica RM2245) and stained using haematoxylin and eosin. The measurements of the diameter of the renal corpuscle (RC), glomeruli (GM), and Bowman’s space (BS) were made using a light microscope (Olympus BX41), a digital microscope camera (5MP binocular microscope, electronic eyepiece USB video CMOS camera, and TopView Image Analyzer). Six GM were measured for each animal, and an average was taken for each individual.

Arteriolosclerosis was graded as described by Sommers et al. [40], where grade 0 indicates unaltered arterioles, grade 1 describes minor localised thickening, grade 2 represents a thickened wall equal to the diameter of the lumen, and grade 3 describes arterioles with wall thickness that exceeded the diameter of the lumen. Hyaline droplets were assessed as number per 50 high-power fields (HPFs). All Hematoxylin -Eosin (HE)-stained sections from both immersion and perfusion samples were examined histopathologically, with special regard to hyaline droplets and eosinophilic bodies. 

### 2.11. Statistics

Statistical analyses were performed using SPSS (Version 16; IBM Corp., Armonk, NY, USA). Data are expressed as the mean (M) ± standard deviation (SD). Normal distribution was assessed using the Shapiro–Wilk test. One-way Analysis of variance (ANOVA) and Least Significant Difference (LSD) post hoc tests were used to assess significant differences between the means of the measured parameters (i.e., oxidative stress, HbA1c, serum lipids, urea, and creatinine) in the studied groups. A *p*-value of <0.05 was considered to be statistically significant.

## 3. Results

By measuring HbA1c, the overall picture of average blood sugar levels can be determined over a period of weeks/months. The results showed that the levels of HbA1c were significantly higher in untreated diabetic rats (9.0% ± 0.7%) than in control rats (4.5% ± 0.5%, *p* < 0.001), indicating that DM was successfully induced by STZ in Sprague-Dawley rats at a dose of 65 mg/kg of body weight (Table 1).

Diabetic rats exhibited higher levels of lipid peroxidase (17.5 ± 1.03 nM) and NO (18.4 ± 5.1 nM) than control rats (9.5 ± 2.2 nM and 9.3 ± 5.1 nM respectively, *p* ≤ 0.01), indicating that inducing DM in Sprague-Dawley rats resulted in an increase in oxidative capacity (Table 1).

TAC was significantly lower in untreated diabetic rats (0.2 ± 0.04 mM/L) than in the control group (0.8 ± 0.2 mM, *p* < 0.001, Table 1).

There were no significant differences between the urea concentration and lipid profiles of untreated diabetic and control rats. In contrast, creatinine levels were significantly higher in untreated diabetic rats (1.2 ± 0.2 mg/dL) than in control rats (0.9 ± 0.1 mg/dL) (Table 1).

Treating diabetic rats with TQ (at a dose of 50 mg/kg body weight) significantly decreased the levels of HbA1c (6.7% ± 1.0%), lipid peroxidase (10.6 ± 2.3 nM), and NO (12.5 ± 2.98 nM) in comparison to untreated diabetic rats (9.0% ± 0.7%, 17.5 ± 1.02, and 18.4 ± 5.1 respectively, *p* < 0.05, Table 1). In addition, the finding that TAC was significantly higher in diabetic rats treated with TQ (0.6 ± 0.2 mM) than in untreated diabetic rats (0.2 ± 0.04 mM, *p* = 0.001) indicates that TQ enhances antioxidant defence mechanisms. Interestingly, a comparison of parameters related to oxidative stress levels of lipid peroxidase and NO between diabetic rats treated with TQ and control rats revealed that there were no statistically significant differences between the two groups (*p* > 0.05). Furthermore, the total cholesterol and urea levels of untreated diabetic rats and diabetic rats treated with TQ were nearly similar to those of the control group (Table 1).

**Figure 1:**
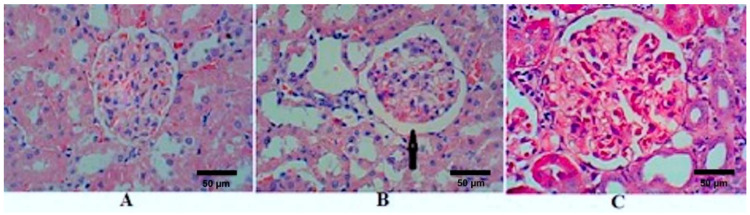
Effects of streptozotocin (STZ) and TQ on glomerular morphology. (**A**) Control group (Group I), (**B**) untreated diabetic group (Group II), and (**C**) TQ-treated diabetic group (Group III). The black arrow indicates an increase in Bowman’s space. H&E staining, magnification = 400 X.

**Figure 2:**
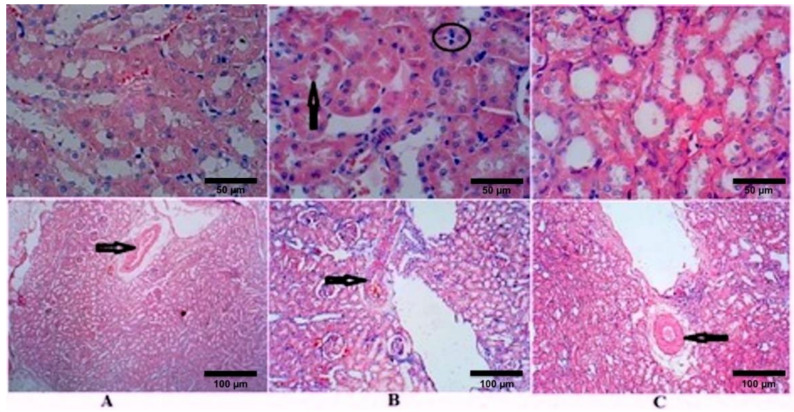
Effects of streptozotocin and TQ on tubulointerstitial morphology (upper images) and on arteriolosclerosis (lower images). (**A**) Control group (Group I), (**B**) untreated diabetic group (Group II), and (**C**) TQ-treated diabetic group (Group III). The vertical black arrow indicates hyaline droplets, inflammatory cells are enclosed in a black circle, and arterioles are indicated by horizontal black arrows. H&E stain; magnification = 400 X in the upper images and 100 X in the lower images.

Kidney sections stained with HE of the control rats (group I) showed normal GM (Figure 1A). After 4 weeks of STZ treatment, sections showed an increase in the diameter of RC, GM, and BS in the untreated diabetic rats (group II); however, GM did not achieve a statistical difference (Figure 1B and Table 2). There were reversible changes after the treatment with TQ (group III), which showed a significant reduction in RC, GM, and BS (Figure 1C). The diameters of RC, GM, and BS of TQ-treated rats decreased and became comparable with the control group (Table 2). Figure 2 and Table 2 show the effects of STZ and TQ on tubulointerstitial morphology (upper image) and arteriolosclerosis (lower images). Control group shows normal tubulointerstitial morphology and grade 0 arteriolosclerosis (Figure 1A). The inflammatory cell infiltration and hyaline droplets in untreated (group II) and TQ-treated (group III) diabetic rats are shown in Figure 1B and C, respectively. The vertical black arrow indicates hyaline droplets, inflammatory cells are enclosed in a black circle, and arterioles are indicated by horizontal black arrows. Arteriolosclerosis was observed in the untreated diabetic group in most of the examined fields, where wall diameter exceeded that of the lumen in nearly all sclerotic arterioles. However, the degree of sclerosis was markedly reduced in TQ-treated diabetic rats. The renal tubules were dilated and hypertrophied in the untreated diabetic group in comparison to the TQ-treated diabetic group (Table 2, Figure 1 and Figure 2).

## 4. Discussion

The present study sought to elucidate the short-term effects of TQ as a safe natural product for treatment and controlling oxidative stress, glycaemic control, lipid profile, and renal functions in diabetic rats. The present study revealed several findings. First, we demonstrated that inducing DM in Sprague-Dawley rats significantly disturbs the normal redox state, as was indicated by altered levels of lipid peroxidase, NO, and TAC in untreated diabetic rats relative to control rats. Similar to the findings of Siboto et al. [41] and Sassy-Prigent et al. [42], histopathology in the present study revealed that injecting rats with STZ resulted in glomerular hypertrophy, which could be largely attributed to mesangial expansion and the thickening of the glomerular basement membrane_._

Another important finding is that treating diabetic rates with TQ successfully improved glycaemic control and attenuated oxidative stress. Interestingly, we observed that the levels of lipid peroxidase, NO, and TAC were similar or better in diabetic rats treated with TQ compared to control rats, indicating that TQ exerts a positive effect on oxidative stress. Additionally, treatment with TQ decreased the diameter of the RC in comparison to that of untreated diabetic rats, while the diameter of BS in diabetic rats treated with TQ decreased even beyond that of control rats. This can be attributed to the capsular thinning and glomerulus preservation observed when comparing the glomerulus diameters in treated and untreated diabetic groups. Ozdemir et al. [43] reported a direct relationship between an increase in the area of BS and blood glucose levels in STZ-induced diabetic nephropathy. The present study demonstrates that treatment with TQ reduces arteriosclerotic changes in the kidney sections taken from treated diabetic rats. Arteriolosclerosis is widely reported in cases of diabetic nephropathy and represents one of the underlying pathophysiological mechanisms of this disease, in addition to oxidative stress and associated inflammation [44].

A study performed by Seyer-Hansen et al. [45] on renal hypertrophy in rats with STZ-induced DM found that the length of renal tubules increased to more than 100 m/kidney and that the diameter of the renal tubular lumen significantly increased 47 days after the onset of DM. Although hyaline droplets are not specific to DM, their presence is largely related to the severity of the disease, as they are directly correlated with the degree of proteinuria [46]. In the present study, treating diabetic rats with TQ reduced the percentage of hyaline droplets by 10%.

The present study also revealed that inducing DM in Sprague-Dawley rats and subsequently treating with TQ did not substantially affect rat lipid profiles over the experimental period. STZ is commonly used to induce DM in rodents on an experimental basis, likely owing to its harmful effects on β-cells in the pancreas [47]. The cytotoxic action of STZ on pancreatic β-cells is likely mediated by enhanced intracellular methylation reactions, DNA strand breaks, and ROS production [48]. However, the molecular mechanisms underlying STZ-induced cell toxicity are still unclear. A recent study conducted by Raza and John [49] investigated STZ-induced cytotoxicity in human hepatoma cells by treating hepatoma cells with different doses of STZ for various time intervals and observing the cytotoxic effects via alterations in redox balance. According to their results, STZ increased levels of oxidative stress, which was evident by the elevated levels of LPO and altered antioxidant capacity. These findings are further supported by the results of the present study, which also observed an increase in the levels of LPO and NO, but a decrease in TAC following the administration of STZ to Sprague-Dawley rats.

It is evident that the disruption of redox balance following the administration of STZ to the Sprague-Dawley rats in the present study was successful in establishing DM and compromised renal function. Altered renal function, as indicated by the significantly higher creatinine levels in untreated diabetic rats in comparison to control rats, is likely the result of a disruption to redox balance in the renal cortex [48] and/or increased renal vascular resistance [50,51]. In the early 2000s, Jacheć et al. [52] investigated disturbances in redox balance in the renal cortex of rats over the course of experimental diabetes. The results of that study were comparable to the findings of the current study in that they observed that plasma creatinine was significantly elevated following the induction of diabetes by STZ. 

It has been previously shown that STZ-induced DM is likely to induce an atherogenic pattern of dyslipidaemia [52,53]. Hypercholesterolemia in STZ-induced diabetic rats usually results from enhanced intestinal absorption and an increase in cholesterol synthesis [54]. In the current study, changes in the levels of total cholesterol, LDL, and HDL were characteristic of an atherogenic pattern of dyslipidaemia; however, most of these lipids were not significantly different between untreated diabetic rats and the control group. In addition, the present study found that HbA1c levels were significantly lower following the treatment of STZ-induced diabetic rats with TQ, while the levels of lipid peroxidase, NO, and TAC were better than untreated diabetic rats. These findings provide further confirmation of the potential beneficial effects of TQ on glycaemic control [55,56] and oxidative stress [57,58], despite the fact that most previous studies were mainly conducted using the plant *N*. *sativa* rather than the active ingredient TQ.

Fararh et al. [11] examined the effect of TQ on glycaemic control and energy metabolism-related enzymes in the leukocytes of STZ-induced diabetic rats and reported significantly lower plasma glucose levels following treatment with TQ. The suggested mechanisms of action of TQ in lowering glucose are thought to be increased insulin levels and the enhanced activities of some cytosolic and mitochondrial enzymes. These findings are further supported by Pari and Sankaranarayanan et al. [59], who showed that TQ therapy was associated with beneficial changes in hepatic enzyme activities and subsequently in hypoglycaemic effects. The lower levels of HbA1c in diabetic rats treated with TQ may also be enhanced by the increased turnover of red blood cells (RBCs) [60,61,62]. This is because the decrease in HbA1c from the rapid turnover of RBCs is likely to be lower than that of HbA1c formation owing to the improved glycaemic control induced by TQ.

Although previous studies have demonstrated the hypolipidemic effect of TQ [59], the present study and several other previous studies failed to prove this effect. Interestingly, an older study by Bamosa et al. [63] conducted in healthy humans showed that while the ingestion of powdered *N. sativa* for two weeks was associated with a significant decrease in plasma cholesterol level in the first week, this parameter paradoxically increased to original levels by the end of the second week. Although the findings of Bamosa et al. are difficult to interpret, they are in agreement with the present results, which failed to demonstrate a significant effect of TQ treatment on the lipid profile of diabetic rats after four weeks. Our findings with regard to the effect of TQ on the lipid profile are supported not only by Bamosa et al., but also by a randomised, double-blind trial conducted by Qidwai et al. [64], which assessed the effectiveness of powdered *N. sativa* in lowering the serum lipid profile. The results of the study by Qidwai et al. failed to demonstrate a statistically significant favourable impact of *N. sativa* on the levels of triglycerides, total cholesterol, HDLs, or LDLs. Based on previous reports and the current study, it may be hypothesised that TQ treatment decreases the lipid profile for only a short duration but has no long-term effects, likely owing to desensitisation mechanisms. Further studies are needed to compare the short- and long-term effects of TQ treatment on serum lipid profiles.

A potential limitation of the present study is that serum insulin and C-peptide levels were not assessed. Further studies that correlate the attenuation in oxidative stress with improved levels of insulin or C-peptide following treatment with TQ would support the present study to provide a more complete picture of the effectiveness of this promising treatment. Another limitation of the present study is that the pancreas, liver, kidney, and respiratory system were not examined histologically to exclude any possible toxic effects of TQ treatment on these organs. Noteworthy, the rats treated with TQ were exposed to DMSO, but not the other studied groups. Theoretically speaking, DSMO may act as a confounding factor for the observed effects of TQ in the present study. Investigators in the field are requested to consider this potential limitation in future studies. Addition of a fourth vehicle (DSMO) group of rates to the study protocol will help to explore the possible effects of DSMO on the studied variables.

In conclusion, the results of the present study demonstrate the potential therapeutic effects of TQ in lowering BGL and attenuating oxidative stress. Moreover, treatment with TQ at a dose of 50 mg/kg body weight exerted no detectable detrimental effects on renal function in rats; however, further studies are needed to adjust the optimal dose for future human studies. Although TQ exhibited a protective effect on glomeruli by reducing BS, and opposed the diabetic effect of STZ, the underlying mechanism for the observed increase in the diameter of the GM in diabetic rats treated with TQ is unclear. While phenomena such as mesangial proliferation, capillary proliferation, or oedema could be the leading cause of this effect, further investigation will be necessary to elucidate the molecular mechanisms at work.

## Figures and Tables

**Table 1:** Comparison of glycaemic control, oxidative stress, lipid profile, and renal function between the studied groups.

	Control Group M ± SD	Untreated Diabetic Group M ± SD	TQ Treated Diabetic Group M ± SD	F	*p*
**Glycaemic Control**					
-HbA1c	4.5 ± 0.5	9.0 ± 0.7 *	6.7 ± 1.0 *#	14.68	<0.001
**Oxidative Stress**					
-Lipid Peroxidase (nM)	9.5 ± 2.2	17.5 ± 1.03 *	10.6 ± 2.3 #	15.29	<0.001
-NO (nmol/mL)	9.3 ± 5.1	18.4 ± 5.1 *	12.5 ± 2.98 #	7.16	0.001
-TAC (mM/L)	0.8 ± 0.2	0.2 ± 0.04 *	0.6 ± 0.2 *#	26.14	<0.001
**Lipid Profile**					
-Total Cholesterol (mg/dL)	112.2 ± 3.2	117.5 ± 13.3	113.3 ± 6.7	0.97	0.441
-Triglycerides (mg/dL)	124.6 ± 2.1	108.8 ± 17.7	113.7 ± 4.4 *	3.40	0.024
-HDL (mg/dL)	46.9 ± 11.1	40.4 ± 6.4	32.3 ± 8.9 *	4.44	0.008
-LDL (mg/dL)	40.4 ± 11.3	55.3 ± 13.5 *	58.2 ± 4.8 *	10.17	<0.001
**Renal Function**					
-Urea (mg/dL)	20.2 ± 2.0	23.7 ± 6.9	27.7 ± 3.8 *	0.95	0.454
-Creatinine (mg/dL)	0.9 ± 0.1	1.2 ± 0.2	1.1 ± 0.2	2.66	0.056

Abbreviations: NO = nitric oxide, TAC = total antioxidant capacity, HbA1c = glycated haemoglobin, HDL = high-density lipoprotein, LDL = low-density lipoprotein. F = the ratio of the between group variance to the within group variance. *p* = *p*-value, * significantly different compared with group I, # significantly different compared with group II. M ± SD = mean ± standard deviation.

**Table 2:** Diameters of the glomerulus, Bowman’s space, and the renal corpuscle, and an assessment of arteriolosclerosis, inflammatory cells, and hyaline droplets.

	Control Group M ± SD	Untreated Diabetic Group M ± SD	TQ Treated Diabetic Group M ± SD	F	*p*
Diameter of RC (µm)	193.48 ± 20.12	228.08 ± 23.42 *	216.95 ± 11.56	3.44	0.077
Diameter GM (µm)	158.10 ± 19.98	170.00 ± 22.81	184.40 ± 6.92	2.15	0.172
Diameter of BS (µm)	22.10 ± 4.29	30.93 ± 4.96 *	16.23 ± 1.71 #	14.33	0.002
Arteriolosclerosis	Grade 0	Grade 3	Grade 1		
Inflammatory cell infiltration	Insignificant	Mild	Mild		
Hyaline droplets	Insignificant	30%	20%		

F = the ratio of the between group variance to the within group variance. *p* = *p*-value, * significantly different compared with group I, # significantly different compared with group II. M ± SD = mean ± standard deviation.

## Data Availability

The data supporting the present findings are contained within the manuscript.

## Sample Availability

Samples of the compounds are available from the corresponding authors.

## Abbreviations

BGL	blood glucose level
BS	Bowman’s space
DM	diabetes mellitus
DMSO	dimethyl sulfoxide
GM	glomeruli
H_2_O_2_	hydrogen peroxide
H_3_PO_4_	phosphoric acid
HDL	high-density lipoprotein
HbA1c	glycated haemoglobin
LDL	low-density lipoprotein
LPO	lipid peroxidation
M	mean
MDA	malondialdehyde
NO	nitric oxide
RBC	red blood cells
RC	renal capsule
ROS	reactive oxygen species
SD	standard deviation
SPSS	Statistical Package for the Social Sciences
STZ	streptozotocin
TAC	total antioxidant capacity
TBA	thiobarbituric acid
VLDL	very low-density lipoprotein

## References

[1] Grindel A., Guggenberger B., Eichberger L., Pöppelmeyer C., Gschaider M., Tosevska A., Mare G., Briskey D., Brath H., Wagner K.-H. (2016). Oxidative Stress, DNA Damage and DNA Repair in Female Patients with Diabetes Mellitus Type 2. PLoS ONE.

[2] Wolff S.P., Dean R.T. (1987). Glucose autoxidation and protein modification. The potential role of “autoxidative glycosylation” in diabetes. Biochem. J..

[3] Groeneveld O.N., Kappelle L.J., Biessels G.J. (2016). Potentials of incretin-based therapies in dementia and stroke in type 2 diabetes mellitus. J. Diabetes Investig..

[4] Mullarkey C.J., Edelstein D., Brownlee M. (1990). Free radical generation by early glycation products: A mechanism for accelerated atherogenesis in diabetes. Biochem. Biophys. Res. Commun..

[5] Besseiche A., Riveline J.-P., Delavallée L., Foufelle F., Gautier J.-F., Blondeau B. (2018). Oxidative and energetic stresses mediate beta-cell dysfunction induced by PGC-1α. Diabetes Metab..

[6] Mooradian A.D. (2009). Dyslipidemia in type 2 diabetes mellitus. Nat. Clin. Pract. Endocrinol. Metab..

[7] Yuan C., Lai C.W.K., Chan L.W.C., Chow M., Law H.K.W., Ying M. (2014). Cumulative effects of hypertension, dyslipidemia, and chronic kidney disease on carotid atherosclerosis in Chinese patients with type 2 diabetes mellitus. J. Diabetes Res..

[8] Giacco F., Brownlee M. (2010). Oxidative Stress and Diabetic Complications. Circ. Res..

[9] Diwan V., Gobe G., Brown L. (2014). Glibenclamide improves kidney and heart structure and function in the adenine-diet model of chronic kidney disease. Pharmacol. Res..

[10] Hong J., Zhang Y., Lai S., Lv A., Su Q., Dong Y., Zhou Z., Tang W., Zhao J., Cui L. (2013). Effects of Metformin Versus Glipizide on Cardiovascular Outcomes in Patients With Type 2 Diabetes and Coronary Artery Disease. Diabetes Care.

[11] Fararh K.M., Ibrahim A.K., Elsonosy Y.A. (2010). Thymoquinone enhances the activities of enzymes related to energy metabolism in peripheral leukocytes of diabetic rats. Res. Vet. Sci..

[12] Jung M., Park M., Lee H., Kang Y.-H., Kang E., Kim S. (2006). Antidiabetic Agents from Medicinal Plants. Curr. Med. Chem..

[13] Jalili C., Salahshoor M.R., Hoseini M., Roshankhah S., Sohrabi M., Shabanizadeh A. (2017). Protective Effect of Thymoquinone Against Morphine Injuries to Kidneys of Mice. Iran J. Kidney Dis..

[14] Arumugam P., Subramanian R., Priyadharsini J.V., Gopalswamy J. (2016). Thymoquinone inhibits the migration of mouse neuroblastoma (Neuro-2a) cells by down-regulating MMP-2 and MMP-9. Chin. J. Nat. Med..

[15] Darakhshan S., Bidmeshki Pour A., Hosseinzadeh Colagar A., Sisakhtnezhad S. (2015). Thymoquinone and its therapeutic potentials. Pharmacol. Res..

[16] Magdy M.-A., Hanan E.-A., Nabila E.-M. (2012). Thymoquinone: Novel gastroprotective mechanisms. Eur. J. Pharmacol..

[17] Sankaranarayanan C., Pari L. (2011). Thymoquinone ameliorates chemical induced oxidative stress and β-cell damage in experimental hyperglycemic rats. Chem. Biol. Interact.

[18] Kanter M. (2009). Protective effects of thymoquinone on streptozotocin-induced diabetic nephropathy. J. Mol. Histol..

[19] Fararh K.M., Shimizu Y., Shiina T., Nikami H., Ghanem M.M., Takewaki T. (2005). Thymoquinone reduces hepatic glucose production in diabetic hamsters. Res. Vet. Sci..

[20] Mathur M.L., Gaur J., Sharma R., Haldiya K.R. (2011). Antidiabetic Properties of a Spice Plant Nigella sativa. J. Endocrinol. Metab..

[21] Megantara S., Utami D., Puspitasari L., Mustarichie R. (2018). In silico study of thymoquinone as peroxisome proliferator activated receptor gamma agonist in the treatment of type 2 diabetes mellitus. J. Biomol. Struct. Dyn..

[22] Al-Ali A., Alkhawajah A.A., Randhawa M.A., Shaikh N.A. (2008). Oral and intraperitoneal LD50 of thymoquinone, an active principle of Nigella sativa, in mice and rats. J. Ayub. Med. Coll. Abbottabad..

[23] Asgary S., Sahebkar A., Goli-Malekabadi N. (2015). Ameliorative effects of Nigella sativa on dyslipidemia. J. Endocrinol. Investig..

[24] Juul S., Poulsen S., Lunn S., Sørensen P., Jakobsen J.C., Simonsen S. (2019). Short-term versus long-term psychotherapy for adult psychiatric disorders: A protocol for a systematic review with meta-analysis and trial sequential analysis. Syst. Rev..

[25] Tawfeek H.M., Abdellatif A.A.H., Dennison T.J., Mohammed A.R., Sadiq Y., Saleem I.Y. (2017). Colonic delivery of indometacin loaded PGA-co-PDL microparticles coated with Eudragit L100-55 from fast disintegrating tablets. Int. J. Pharm..

[26] Demontis F., Loi F., Malesa R., D’Aquila P.S., Serra G. (2011). Antidepressant-like effect of cannabiniod CB1 receptor stimulation in the forced swimming test. Int. Clin. Psychopharmacol..

[27] Masiello P., Broca C., Gross R., Roye M., Manteghetti M., Hillaire-Buys D., Novelli M., Ribes G. (1998). Experimental NIDDM: Development of a new model in adult rats administered streptozotocin and nicotinamide. Diabetes.

[28] Manabe Y., Tochigi M., Moriwaki A., Takeuchi S., Takahashi S. (2013). Insulin-like growth factor 1 mRNA expression in the uterus of streptozotocin-treated diabetic mice. J. Reprod. Dev..

[29] RILEY V. (2017). Adaptation of orbital bleeding technic to rapid serial blood studies. Proc. Soc. Exp. Biol. Med..

[30] Bunn H.F., Haney D.N., Kamin S., Gabbay K.H., Gallop P.M. (1976). The biosynthesis of human hemoglobin A1c. Slow glycosylation of hemoglobin in vivo. J. Clin. Invest.

[31] Ohkawa H., Ohishi N., Yagi K. (1979). Assay for lipid peroxides in animal tissues by thiobarbituric acid reaction. Anal. Biochem..

[32] Koracevic D., Koracevic G., Djordjevic V., Andrejevic S., Cosic V. (2001). Method for the measurement of antioxidant activity in human fluids. J. Clin. Pathol..

[33] Miranda K.M., Espey M.G., Wink D.A. (2001). A Rapid, Simple Spectrophotometric Method for Simultaneous Detection of Nitrate and Nitrite. Nitric Oxide.

[34] Fabiny D.L., Ertingshausen G. (1971). Automated reaction-rate method for determination of serum creatinine with the CentrifiChem. Clin. Chem..

[35] Tabacco A., Meiattini F., Moda E., Tarli P. (1979). Simplified enzymic/colorimetric serum urea nitrogen determination. Clin. Chem..

[36] Fossati P., Prencipe L. (1982). Serum triglycerides determined colorimetrically with an enzyme that produces hydrogen peroxide. Clin. Chem..

[37] Allain C.C., Poon L.S., Chan C.S., Richmond W., Fu P.C. (1974). Enzymatic determination of total serum cholesterol. Clin. Chem..

[38] Warnick G.R., Albers J.J. (1978). A comprehensive evaluation of the heparin-manganese precipitation procedure for estimating high density lipoprotein cholesterol. J. Lipid Res..

[39] Nauck M., Warnick G.R., Rifai N. (2002). Methods for measurement of LDL-cholesterol: A critical assessment of direct measurement by homogeneous assays versus calculation. Clin. Chem..

[40] Morales A.I., Detaille D., Prieto M., Puente A., Briones E., Arévalo M., Leverve X., Lopez-Novoa J.M., El-Mir M.-Y. (2010). Metformin prevents experimental gentamicin-induced nephropathy by a mitochondria-dependent pathway. Kidney Int..

[41] Siboto A., Sibiya N., Khathi A., Ngubane P. (2018). The effects of Momordica balsamina methanolic extract on kidney function in STZ-induced diabetic rats: Effects on selected metabolic markers. J. Diabetes Res..

[42] Sassy-Prigent C., Heudes D., Mandet C., Bélair M.F., Michel O., Perdereau B., Bariety J., Bruneval P. (2000). Early glomerular macrophage recruitment in streptozotocin-induced diabetic rats. Diabetes..

[43] Al Dubayee M.S., Alayed H., Almansour R., Alqaoud N., Alnamlah R., Obeid D., Alshahrani A., Zahra M.M., Nasr A., Al-Bawab A. (2018). Differential Expression of Human Peripheral Mononuclear Cells Phenotype Markers in Type 2 Diabetic Patients and Type 2 Diabetic Patients on Metformin. Front. Endocrinol..

[44] Malik S., Suchal K., Khan S.I., Bhatia J., Kishore K., Dinda A.K., Arya D.S. (2017). Apigenin ameliorates streptozotocin-induced diabetic nephropathy in rats via MAPK-NF-κB-TNF-α and TGF-β1-MAPK-fibronectin pathways. Am. J. Physiol..

[45] Seyer-Hansen K., Hansen J., Gundersen H.J. (1980). Renal hypertrophy in experimental diabetes. A morphometric study. Diabetologia.

[46] Motshakeri M., Ebrahimi M., Goh Y.M., Othman H.H., Hair-Bejo M., Mohamed S. (2014). Effects of Brown Seaweed (Sargassum polycystum) Extracts on Kidney, Liver, and Pancreas of Type 2 Diabetic Rat Model. Evid. Based. Complement. Alternat. Med..

[47] King A.J. (2012). The use of animal models in diabetes research. Br. J. Pharmacol..

[48] Rao V.S.N., Santos F.A., Silva R.M., Teixiera M.G. (2002). Effects of nitric oxide synthase inhibitors and melatonin on the hyperglycemic response to streptozotocin in rats. Vascul. Pharmacol..

[49] Raza H., John A. (2012). Streptozotocin-Induced Cytotoxicity, Oxidative Stress and Mitochondrial Dysfunction in Human Hepatoma HepG2 Cells. Int. J. Mol. Sci..

[50] Zhao H.J., Wang S., Cheng H., Zhang M., Takahashi T., Fogo A.B., Breyer M.D., Harris R.C. (2006). Endothelial nitric oxide synthase deficiency produces accelerated nephropathy in diabetic mice. J. Am. Soc. Nephrol..

[51] Li W., Wang G., Lu X., Jiang Y., Xu L., Zhao X. (2014). Lycopene ameliorates renal function in rats with streptozotocin-induced diabetes. Int. J. Clin. Exp. Pathol..

[52] Jacheć W., Tomasik A., Tarnawski R., Chwalińska E. (2002). Evidence of oxidative stress in the renal cortex of diabetic rats: Favourable effect of vitamin E. Scand J. Clin. Lab. Invest..

[53] Kim A.-R., Jeong S.-M., Kang M.-J., Jang Y.-H., Choi H.-N., Kim J.-I. (2013). Lotus leaf alleviates hyperglycemia and dyslipidemia in animal model of diabetes mellitus. Nutr. Res. Pract..

[54] Raghunathan S., Tank P., Bhadada S., Patel B. (2014). Evaluation of buspirone on streptozotocin induced type 1 diabetes and its associated complications. Biomed. Res. Int..

[55] Mathé D. (1995). Dyslipidemia and diabetes: Animal models. Diabetes Metab..

[56] Bamosa A.O., Kaatabi H., Lebdaa F.M., Elq A.-M., Al-Sultanb A. (2010). Effect of Nigella sativa seeds on the glycemic control of patients with type 2 diabetes mellitus. Indian J. Physiol. Pharmacol..

[57] Bamosa A., Lebda F., Al Elq A., Al-Sultan A., Kaatabi H. (2012). Favorable impact of Nigella sativa seeds on lipid profile in type 2 diabetic patients. J. Fam. Community Med..

[58] Ahmad S., Beg Z.H. (2014). Mitigating role of thymoquinone rich fractions from Nigella sativa oil and its constituents, thymoquinone and limonene on lipidemic-oxidative injury in rats. Springerplus.

[59] Pari L., Sankaranarayanan C. (2009). Beneficial effects of thymoquinone on hepatic key enzymes in streptozotocin-nicotinamide induced diabetic rats. Life Sci..

[60] Asgary S., Najafi S., Ghannadi A., Dashti G., Helalat A. (2012). Efficiency of black cumin seeds on hematological factors in normal and hypercholesterolemic rabbits. ARYA Atheroscler..

[61] Ahmad A., Asif H., Mohd M., Alam K.S., Kalam N.A., Ali S.N., Damanhouri Z.A., Anwar F. (2013). A review on therapeutic potential of Nigella sativa: A miracle herb. Asian Pac. J. Trop. Biomed..

[62] Meral I., Kanter M. (2003). Effects of *Nigella sativa* L. and *Urtica dioica* L. on Selected Mineral Status and Hematological Values in CCl4-Treated Rats. Biol. Trace Elem. Res..

[63] Bamosa A.O., Ali B.A., Sowayan S.A. (1997). Effect of oral ingestion of Nigella sativa seeds on some blood parameters. Saudi Pharm. J..

[64] Qidwai W., Hamza H.B., Qureshi R., Gilani A. (2009). Effectiveness, safety, and tolerability of powdered Nigella sativa (ka-lonji) seed in capsules on serum lipid levels, blood sugar, blood pressure, and body weight in adults: Results of a ran-domized, double-blind controlled trial. J. Altern. Complementary Med..

